# Association between Environmental Temperature and Survival in Gastroesophageal Cancers: A Population Based Study

**DOI:** 10.3390/cancers16010074

**Published:** 2023-12-22

**Authors:** Kush Gupta, Anthony George, Kristopher Attwood, Ashish Gupta, Arya Mariam Roy, Shipra Gandhi, Beas Siromoni, Anurag Singh, Elizabeth Repasky, Sarbajit Mukherjee

**Affiliations:** 1 Department of Internal Medicine, Umass Chan Medical School—Baystate, Springfield, MA 01199, USA; kush.gupta@baystatehealth.org; 2 Department of Biostatistics and Bioinformatics, Roswell Park Comprehensive Cancer Center, Buffalo, NY 14263, USAkristopher.attwood@roswellpark.org (K.A.); 3 Department of Hematology and Oncology, Roswell Park Comprehensive Cancer Center, Buffalo, NY 14263, USAarya.roy@roswellpark.org (A.M.R.);; 4 School of Health Sciences, University of South Dakota, Vermillion, SD 57069, USA

**Keywords:** esophageal cancer, gastric cancer, environmental temperature, cold stress, overall survival, disease-specific survival

## Abstract

**Simple Summary:**

Recent animal studies have shown a correlation between environmental temperature and tumor growth. Based on these studies, we hypothesized that esophageal cancer and gastric cancer patients living in warmer climates have improved survival as compared to patients living in colder climates. We conducted a study using the SEER (Surveillance, Epidemiology, and End Results) database and analyzed the cancer outcomes with the county-level average annual temperature in which those patients resided. We analyzed 17,408 esophageal cancer and 20,533 gastric cancer patients. We noted for the first time that higher environmental temperatures were associated with significant improvements in survival in patients with esophageal and gastric cancers. Further confirmatory population-based studies as well as mechanistic-bench studies are needed to support our findings.

**Abstract:**

Background: Cold stress suppresses antitumor response in animal models, leading to tumor growth. Recent studies have also shown a negative correlation between the average annual temperature (AAT) and cancer incidence. We hypothesized that esophageal cancer (EC) and gastric cancer (GC) patients living in warmer climates have improved survival outcomes than those living in colder climates. Methods: We conducted a retrospective analysis using the Surveillance, Epidemiology, and End Results (SEER) database from 1996 to 2015. We retrieved the National Centers for Environmental Information data to calculate the county-level AAT. Cox multivariate regression models were performed to measure the association between temperature (measured continuously at diagnosis and in 5-degree increments) and OS/DSS, adjusting for variables. All associations were compared at a significance level of 0.05. The OS and DSS were summarized using Kaplan–Meier methods. All statistics were performed using SAS version 9.4 (SAS Institute Inc., Cary, NC, USA). Results: A total of 17,408 EC patients were analyzed. The average age of the cohort was 65 years, 79% of which were males and 21% were females. Of them, 61.6% had adenocarcinoma, and 37.6% were squamous. After adjusting for covariates, patients in regions with an AAT > 53.5 °F had an 11% improvement in OS [HR 0.89 (95% CI 0.86–0.92), *p* < 0.0001] and 13% in DSS [HR 0.87 (95% CI 0.84–0.90), *p* < 0.0001]. When the temperature was analyzed in 5 °F increments, with each increment, there was a 3% improvement in OS [HR 0.97 (95% CI 0.96–0.98), *p* < 0.0001] and 4% in DSS [HR 0.96 (95% CI 0.95–0.97), *p* < 0.0001]. Subgroup analysis of squamous and adenocarcinoma showed similar results. These findings were validated in 20,553 GC patients. After adjusting for covariates, patients in regions with an AAT > 53.5 had a 13% improvement in OS [HR 0.87 (95% CI 0.85–0.90), *p* < 0.0001] and 14% in DSS [HR 0.86 (95% CI 0.83–0.89), *p* < 0.0001]. When analyzed in 5 °F increments, with each increment, there was a 4% improvement in OS [HR 0.96 (95% CI 0.952–0.971), *p* < 0.0001] and 4% in DSS [HR 0.96 (95% CI 0.945–0.965), *p* < 0.0001]. Conclusion: We showed for the first time that higher environmental temperatures are associated with significant improvements in OS and DSS in patients with gastro-esophageal cancers, notwithstanding the limitations of a retrospective database analysis. Further confirmatory and mechanistic studies are required to implement specific interventional strategies.

## 1. Introduction

Gastroesophageal cancers (GEC) are highly aggressive malignancies and leading causes of cancer-related mortality. According to GLOBOCAN 2020 statistics, esophageal (EC) and gastric cancers (GC) were the fourth and sixth leading causes of cancer-related deaths globally, with 768,793 and 544,076 new deaths reported, respectively [1]. In the United States (US), the estimated annual new cases of EC and GC were 26,380 and 20,640, respectively, in 2022 [2]. Unfortunately, majority of the patients are diagnosed at advanced stages, and the prognosis of EC and GC remains poor (5-year overall survival [OS] in 2022 ranged between 20–32%) [3,4,5]. In addition, patients with GEC may suffer from a wide range of morbidities, such as bleeding, obstruction, and worsened quality of life [6,7]. Therefore, GECs are a global public health concern that substantially burdens patients and healthcare resource utilization.

Several environmental risk factors are implicated in developing GEC, which vary according to the underlying histology and biological characteristics. Tobacco smoking and alcohol consumption are predominantly associated with esophageal squamous cell carcinoma, while gastroesophageal reflux, obesity, and low fruit/vegetable intake are associated with the esophageal adenocarcinoma subtype [8]. Additionally, *H. pylori* infection, Epstein–Barr virus (EBV), and dietary factors increase the risk of GC [9,10]. Several prognostic factors affect survival in GECs, such as advanced age, location, histological type, stage and lymph node status, and genetic biomarkers [11,12,13,14]. However, patient, disease, and management-specific factors were not found to fully explain the geographical disparities of GE outcomes [15,16]. Therefore, further research is needed to evaluate potential environmental and social prognostic factors for GEC.

Acute and chronic stressors are well-established modulators of the tumor microenvironment and significantly promote cancer invasion and progression through cascades of signaling pathways and adaptive immune responses [17]. Chronic cold stress has been found to prompt genetic and pre-genetic alterations and immunosuppressive responses, creating a pro-tumorigenic microenvironment [18,19]. Previous animal models have shown that chronic cold temperatures alter murine physiology and dysregulate immune response, leading to overexpression of immunosuppressive M2 macrophages, excessive release of pro-inflammatory cytokines and regulatory T cells (Tregs), and myeloid-derived suppressor cells (MDSCs)-mediated suppression of immune effector T cells [20,21]. It has also been found that sub-thermoneutral housing temperature (22 °C/71.6 °F) promotes tumor cell proliferation, pro-metastatic effects, and cancer progression [22,23,24].

Such findings led epidemiological studies to investigate the “cancer-cold” hypothesis, a term that denotes higher cancer risk in areas with colder temperatures. In a previous retrospective study, patients from the coldest countries were found to have the highest cancer incidence [25]. Likewise, data from the US found that lower environmental temperature/average annual temperature (AAT) was a significant predictor of higher cancer risk, including GEC and EC [26,27]. Notably, colder temperatures were found to have prognostic implications and significantly affected cancer survival. In a study by Sharma et al., most countries with the coldest AAT were among the top 50 countries with the highest cancer-related mortality, indicating a negative predictive value of colder AAT on cancer outcomes [28]. More recently, it was found that lower environmental temperatures were significantly associated with worse OS in patients with breast cancer [29].

Although there is an increasing body of evidence associating environmental temperature with cancer incidence, there remains a distinct lack of focused research examining the impact of climate on survival outcomes, particularly in GEC patients. Prior research primarily has stratified cancer incidence according to county-specific temperatures, and while it has established a potential relationship between colder temperatures and increased cancer risk, the specific implications of cold temperatures on survival outcomes remain largely unexplored. This study analyzed the data of the Surveillance, Epidemiology, and End Results (SEER) to investigate the predictive value of AAT on the overall (OS) and disease-specific survival (DSS) of GEC patients in the US.

## 2. Materials and Methods

### 2.1. Data Source and Population

The present study was a population-based retrospective analysis that retrieved data of all patients with EC and GE from the SEER database, covering nearly one-third of the US population. Data of all adult patients diagnosed between 1996 and 2017 were retrieved. There were no restrictions regarding the tumor stage. We used the 3rd edition of the WHO International Classification of Diseases for Oncology for tumor site identification. Patients with missing survival follow-up data were excluded. We extracted data regarding demographic characteristics, tumor histology, histological stage, grade and stage, history of surgery, and survival outcomes.

Data regarding the AAT were obtained from the National Centers for Environmental Information (NCEI; https://www.ncei.noaa.gov/, accessed on 20 July 2022), which provides county- and time-specific data regarding the average temperature [30]. We retrieved the county-specific monthly average temperatures at diagnosis to calculate the AAT. As the present study was based on a publicly available de-identified database, the need for ethics committee approval was waived.

### 2.2. Statistical Analysis

All statistical analyses were performed using SAS version 9.4 (SAS Institute Inc., Cary, NC, USA). The mean, median, and standard deviation were reported for continuous variables, with comparisons made using the Kruskal–Wallis and Mann– Whitney U tests. The frequencies and relative frequencies were reported and compared for categorical variables using the chi-square and Fisher’s exact tests. The temperature was treated as continuous, categorized by quantiles (<q1, q1–q3, and >q3), and binary (dichotomized at the optimal cut-point by the maximal log-rank criterion). The OS and DSS were summarized using standard Kaplan–Meier methods. The median survival rates were reported, and log-rank *p*-values were provided. Utilizing Cox univariate regression models, the relationship between AAT (continuously assessed at diagnosis in 5-degree intervals) and OS/DSS was evaluated. The outcomes of this analysis were expressed as hazard ratios (HR) along with their respective 95% confidence intervals (CI). Cox multivariate regression modeling was performed to measure the association between AAT and survival after adjusting for age (continuous), sex, race, stage, histology, and grade. A *p*-value of less than 5% was considered statistically significant.

The relationship between temperature at diagnosis and survival outcomes was assessed for both EC and GC. In addition, a subgroup analysis was conducted according to the histological subtype of malignancy. To validate the findings and compare the survivability between EC and GC, the esophageal cut point was used for secondary analyses of stomach cancer. Survival summaries and results from multivariate analyses were reported.

## 3. Results

A. Patients with esophageal cancer

1. Characteristics of the included patients

A total of 17,408 patients with stage I–IV EC patients from the SEER database were included in the analysis, with a mean age of 65 ± 11.6 years and male predominance (79.4%). Overall, 80% of the patients were non-Hispanic white, and 85.3% lived in urban areas. The most common primary site of malignancy was the lower third of the esophagus (58.7%), followed by the middle third (16.5%). Adenocarcinoma was noted in 61.6% of the patients, while squamous carcinoma was noted in 37.6%. Overall, 41% of the patients had grade I/II disease, and 42% had grade III/IV disease. According to the maximal log-rank criterion, an AAT of 53.5 °F was defined as an optimal cut-off value. Patients living in warmer temperatures (>53.5 °F) had significantly older age (*p* < 0.001) and were more likely to be Hispanic (*p* < 0.001), insured (*p* = 0.037), and higher grade (*p* < 0.001). Table 1 shows the demographic and clinical characteristics of the patients categorized by the AAT.

2. Impact of AAT at diagnosis on survival outcomes

**Figure 1:**
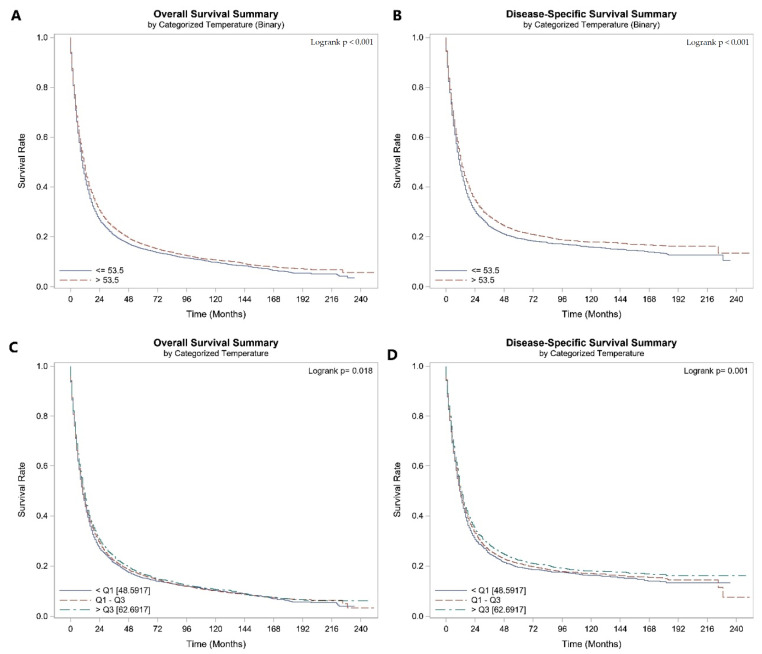
Patients with esophageal cancer: Kaplan–Meier Curve of (**A**) OS of patients living at ATT ≤ 53.5 and >53.5, (**B**) DSS of patients living at ATT ≤ 53.5 and >53.5, (**C**) OS of patients living at different quartiles of ATT, (**D**) DSS of patients living at different quartiles of ATT.

EC patients’ median OS and DSS were 10.0 (95% CI 10.0, 11.0) and 12 (95% CI not defined) months, respectively. Patients living at an AAT > 53.5 °F had significantly longer OS (11 [95% CI not defined] versus 10.0 [95% CI 9.0, 10.0] months; *p* < 0.001) and DSS (13 [95% CI 12, 13] versus 11.0 (95% CI 10.0, 11.0) months; *p* < 0.001) than patients living at a temperature ≤ 53.5 °F (Figure 1A,B). Likewise, when we categorized the AAT according to quantiles, the same findings were observed, where patients living at an AAT > 62.69 °F had longer OS and DSS (Figure 1C,D). The univariate Cox regression showed that, with each 5 °F incremental increase in the AAT, there was a 2% improvement in OS (HR 0.98 [95% CI 0.97–0.99], *p* < 0.001) and 2.6% improvement in DSS (HR 0.974 [95% CI 0.96–0.98], *p* < 0.001). In the adjusted model, a 5 °F incremental increase in the AAT was an independent predictor of OS [HR 0.96 (95% CI 0.95–0.97), *p* < 0.001] and DSS [HR 0.96 (95% CI 0.95–0.97), *p* < 0.001]. The Cox regression model adjusted for covariates showed that living at an AAT > 53.5 °F was an independent predictor of OS (HR 0.89 [95% CI 0.86–0.92], *p* < 0.001) and DSS (HR 0.87 [95% CI 0.84–0.90], *p* < 0.001), Table 2.

In the squamous subgroup, there were 4.1% and 4.2% improvements in OS and DSS, respectively (*p* for adjusted HRs < 0.001) with each 5 °F increment in AAT. The multivariate regression showed that living at an AAT > 53.5 °F was an independent predictor of OS (HR 0.88 [95% CI 0.83–0.92], *p* < 0.001) and DSS (HR 0.87 [95% CI 0.82–0.92], *p* < 0.001), Table 2. The adenocarcinoma subgroup had similar results (*p* < 0.001), with living at an AAT > 53.5 °F was an independent predictor of O (HR 0.90 [95% CI 0.86–0.94], *p* < 0.001) and DSS (HR 0.88 [95% CI 0.84–0.92], *p* < 0.001), Table 2.

B. Gastric cancer patients

1. Characteristics of the included patients

A total of 20,533 patients with GC patients from the SEER database were included in the analysis. The mean age of the study population was 68 years. 67% were male, 58% were non-Hispanic white, 56% were married, and 88% lived in urban areas. 66% were in the stomach, and 34% were at the esophagogastric junction. Within the stomach, cardia was the most common site (34%). Of all patients, 34% had grade I/II disease, and 53% had grade III/IV disease. Patients with stage I–IV cancer were included, with the majority (43%) with stage IV cancer. Patients living in warmer temperatures (>53.5 °F) had significantly older age (*p* < 0.001) and were more likely to be non-Hispanic white (*p* < 0.001) and had a higher grade (*p* < 0.001). Table 3.

2. Impact of AAT at Diagnosis on survival outcomes

**Figure 2:**
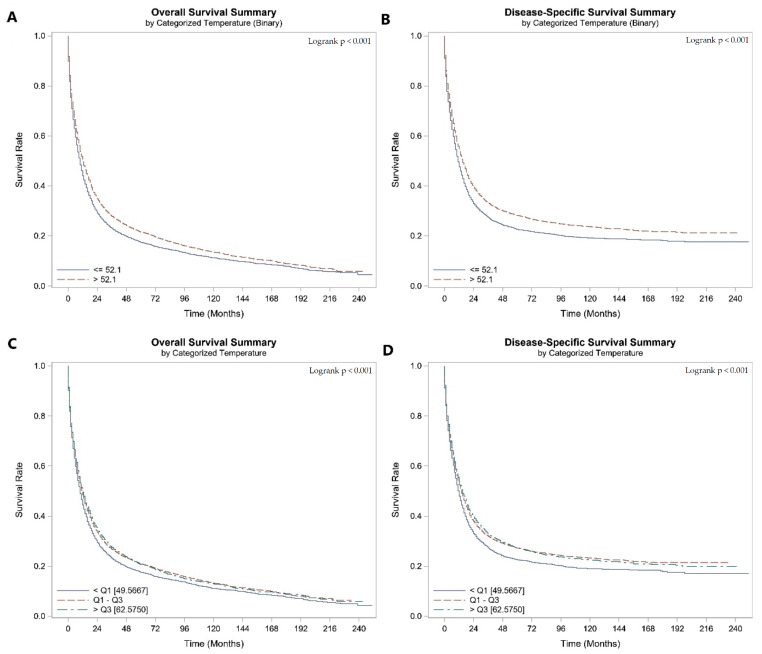
Patients with gastric cancer: Kaplan–Meier Curve of (**A**) OS of patients living at ATT ≤ 53.5 and >53.5, (**B**) DSS of patients living at ATT ≤ 53.5 and >53.5, (**C**) OS of patients living at different quartiles of ATT, (**D**) DSS of patients living at different quartiles of ATT.

The median OS and DSS of GC patients were 11.0 (95% CI 11.0, 12.0) and 13.0 (95% CI 13.0, 14.0) months, respectively. Patients living at an AAT > 53.5 °F had significantly longer OS (13.0 [95% CI 12.0, 13.0] versus 10.0 [95% CI not defined] months; *p* < 0.001) and DSS (15.0 [95% CI 14.0, 16.0] versus 11.0 (95% CI 11.0, 12.0) months; *p* < 0.001) than patients living at a temperature ≤ 53.5 °F (Figure 2A,B). Likewise, when we categorized the AAT according to quantiles, the same findings were observed, where patients living at an AAT > 62.57 °F had longer OS and DSS (Figure 2C,D).

The multivariate Cox regression showed that a 5 °F incremental increase in the AAT was an independent predictor of OS (HR 0.96 [95% CI 0.95–0.97], *p* < 0.001) and DSS (HR 0.96 [95% CI 0.95–0.97], *p* < 0.001). There were a 12.5% improvement in OS (HR 0.88 [95% CI 0.85–0.90], *p* < 0.001) and a 14.2% improvement in DSS (HR 0.86 [95% CI 0.83–0.89], *p* < 0.001) in patients living at an AAT > 53.5 °F, Table 4.

## 4. Discussion

Recent reports have revealed higher cancer incidence and less favorable oncological outcomes in countries with colder climates [26,27]. Interestingly, animal studies at our institution have demonstrated a pro-tumorigenic and metastatic response to sub-thermoneutral temperature in cancer models [23]. In the present population-based study, we demonstrated that higher AAT is associated with more favorable survival outcomes and significantly prolongs the OS and DSS of GEC patients. The results indicated that EC and GC patients had 3.4% and 4% improvements in OS with every 5 °F incremental increase in AAT, respectively. Likewise, EC and GC patients had 4% improvements in DSS with every 5 °F incremental increase in AAT.

The present study’s findings agree with a growing body of evidence suggesting a positive correlation between environmental temperature and survival outcomes of cancer patients. In a recent analysis of 6479 breast cancer patients, Gandhi et al. observed a trend towards worse DSS and OS with a high thermogenesis score, which indicates chronic cold stress [31]. The prognostic value of environmental temperature was also evident in Sharma et al., in which countries with the coldest temperatures had the highest cancer-related mortality rates [28]. Our recent population-based study found that, for every 5 °F incremental increase, there was a 2% improvement in OS in patients with breast cancer [29]. Interestingly, Wang et al. studied the association between latitude and GC clinical outcomes using the Chinese Cancer Genome Atlas database. After adjusting for confounding factors, samples at low latitudes, usually associated with higher temperatures, had a significantly better clinical response and OS than samples at high latitudes [32].

Despite the growing interest in the impact of cold stress or environmental temperatures on the survival of cancer patients, limited data is available to explain the mechanistic pathways that form the basis of the association between cold stress and worse survival. The current literature suggests a significant influence of cold stress on tumor genetics and microenvironment. Past studies have indicated that cold stress induces tumorigenesis by increasing the frequency of somatic mutations [33]. In Wang et al., tumor mutation burden was lower and DNA repair activities were higher in GC samples of patients living at lower latitudes than in patients living at higher latitudes [32]. Cold stress may also alter the tumor microenvironment and host immune response, favoring tumor spread and metastasis. It was found that GC samples from high-latitude regions had a high burden of immune cell infiltration [32]. The cold stress-induced impaired antitumor immune responses were also evident in MacDonald et al., in which sub thermoneutral temperature suppressed functional CD8^+^ T and led to the overexpression of suppressors of antitumor immune responses [19]. In cases with high thermogenesis scores, a typical pro-tumorigenesis/metastatic microenvironment was noted, including upregulated glucocorticoid receptor (GR) signaling pathway, anti-apoptotic activities, lower interferon-gamma (IFN-γ), cytolytic activity, and chemokines-mediated cytotoxic T lymphocytes (CTLs) responses [31,34]. Cold stress was also found to activate programmed death receptor-1 (PD-1), which suppresses cytotoxic T cells and helps tumor cells to evade the immune response [35]. According to Wang et al., patients living at high latitudes had higher expression of the PD-L1 gene, further supporting the potential role of cold stress in impairing antitumor immune responses [32].

Other possible explanations for the negative correlation between temperature and survival in cancer patients include the induction of higher angiogenesis and tumor invasiveness mediated by the adrenergic signaling pathway [31,36,37].

As mentioned above, our results align with previous epidemiological studies showing a significant association between environmental temperatures and the survival of cancer patients. When coupled with experimental evidence demonstrating pro-angiogenic and pro-metastatic responses to sub-thermoneutral housing temperature in cancer models [21], the results of the present study suggest the need to adjust for housing temperature during the evaluation of novel therapies. Kokolus et al. showed that lower housing temperatures (20–26 °C; 68–79 °F) significantly trigger the immunosuppressive microenvironment through overexpression of immunosuppressive cells and suppression of antitumor immune response [23]. On the other hand, tumor bearing mice housed at thermoneutral temperature (30–31 °C; 86–88 °F) had higher antigen specific CD8^+^ T-cells, as well as a reduction in tumor growth rate and metastasis. Housing temperatures may explain the variation in response to immunotherapy in cancer models. Environmental temperature can also be considered a potential confounder in clinical immunotherapy trials. This becomes more relevant in GEC patients due to the ongoing efforts to introduce effective novel immunotherapies for advanced cases.

The results of the present study give rise to the question of whether patients with GEC who live in colder climates could benefit from adjuvant therapies targeting neuronal thermoreceptive pathways. For example, cold stress-induced neuroendocrine activation was found to induce breast cancer spread and metastasis via the β-adrenergic signaling pathway, an effect that was reversed after administrating a non-selective β-blocker [38]. These findings led to early clinical trials investigating add-on non-selective β-blockers in metastatic melanoma and breast cancer, which showed promising antitumor activity and reduced levels of metastatic biomarkers [39,40]. Our team at Roswell Park is using propranolol, a non-selective beta blocker, in two different clinical trials in esophageal cancer to potentially improve antitumor immunity and clinical outcomes (NCT05651594, PI: Mukherjee and NCT04682158, PI: Singh).

Our study is one of the rare population-based reports that evaluated the association between environmental temperatures and the survival of cancer patients. The study retrieved the data of a large cohort of GEC patients from the SEER database, which covers 35% of the US population. In addition, the AAT was based on county-specific data to account for potential variations in the environmental temperatures within the same region. However, we acknowledge the existence of methodological limitations. Firstly, our study is based on a retrospective collection of real-world data from routine clinical practice, which can introduce misclassification and ascertainment biases. The standardization of outcome reporting and definitions was not feasible. The available data limited expanding our adjusted multivariate analysis and accounting for additional confounding factors, such as patients’ response to environmental temperature, tumor markers, chemotherapeutic regimens, or other treatment-specific factors that can impact the survival of GEC patients. Similarly, some other factors such as diet, incidence of *Helicobacter pylori* infection could not be accounted for in the analysis due to unavailability of such data in the SEER database. Healthcare delivery is also impacted by logistic limitations in colder weather. For example, travelling to high-volume and comprehensive cancer centers may be limited in cold weather which may impact the overall outcomes. Compared to individuals living in temperate regions, individuals who live in low-average temperature areas have differences in temperature-dependent host factors, such as their home environment, basic lifestyle, clothing, food, and beverage choices. The composition of their gut microbiome may also be altered [41,42]. Consequently, it may affect their ability to limit tumor development and growth. Due to the unavailability of data regarding the treatment received and its appropriateness, or the environmental temperature-dependent host factors in the SEER database, we could not incorporate these factors into our analysis. We acknowledge the role of such factors in altering survival outcomes. In addition, it was not possible to account for the residency change, in which patients might have been exposed to variable AAT.

In conclusion, the present study suggests a positive impact of higher environmental temperatures on the survival outcomes of patients with GEC. Patients with GEC living in warmer temperatures had a significantly longer OS and DSS, regardless of the pathologic subtype. Despite the methodological limitations implicated in a SEER database analysis, our findings, combined with the previously published animal experiments from our group, highlight the need to consider housing temperatures during the assessment of cancer models and environmental temperature as a potential confounder during the evaluation of the outcomes of GEC patients. In addition, novel therapies targeting neural thermoreceptive pathways may have a role in GEC patients. Future mechanistic studies are warranted to better understand the association between environmental temperature and cancer survival and study the impact of this association on treatment options.

## Figures and Tables

**Table 1:** Characteristics of esophageal cancer cohort according to temperature groups (*n* = 17,408 patients).

		AAT ≤ 53.5 °F	AAT > 53.5 °F	*p*-Value
	N	7637 (43.9)	9771 (56.1)	
Age	Mean/Std/N	64.27/11.52/7637	65.68/11.62/9771	<0.001
Sex	Male	6099 (79.9%)	7719 (79.0%)	0.163
	Female	1538 (20.1%)	2052 (21.0%)	
Race	Non-Hispanic White	6111 (80.0%)	7809 (79.9%)	<0.001
	Non-Hispanic Black	1286 (16.8%)	1420 (14.5%)	
	Hispanic	240 (3.1%)	542 (5.5%)	
Marital Status	Married	4183 (54.8%)	5299 (54.2%)	0.477
	Single	3454 (45.2%)	4472 (45.8%)	
Insurance Status	Insured	3959 (51.8%)	5239 (53.6%)	0.037
	Uninsured	231 (3.0%)	259 (2.7%)	
	Unknown	3447 (45.1%)	4273 (43.7%)	
Primary Site	C150 Cervical esophagus	144 (1.9%)	196 (2.0%)	<0.001
	C151 Thoracic esophagus	264 (3.5%)	353 (3.6%)	
	C152 Abdominal esophagus	47 (0.6%)	82 (0.8%)	
	C153 Upper third of the esophagus	397 (5.2%)	463 (4.7%)	
	C154 Middle third of esophagus	1282 (16.8%)	1593 (16.3%)	
	C155 Lower third of esophagus	4573 (59.9%)	5642 (57.7%)	
	C158 Overlapping lesion of the esophagus	299 (3.9%)	405 (4.1%)	
	C159 Esophagus, NOS	631 (8.3%)	1037 (10.6%)	
Histology	Adenocarcinoma	4768 (62.4%)	5957 (61.0%)	0.104
	Squamous	2811 (36.8%)	3726 (38.1%)	
	Adenosquamous	58 (0.8%)	88 (0.9%)	
Stage	Localized	1931 (25.3%)	2325 (23.8%)	0.076
	Regional	2716 (35.6%)	3539 (36.2%)	
	Distant	2990 (39.2%)	3907 (40.0%)	
Grade	I/II	3195 (41.8%)	3914 (40.1%)	<0.001
	III/IV	3046 (39.9%)	4223 (43.2%)	
	Unknown	1396 (18.3%)	1634 (16.7%)	

°F: Fahrenheit; Std: Standard deviation; NOS: Not otherwise specified.

**Table 2:** Multivariate analysis of AAT as a predictor of OS and DSS in the esophageal cancer cohort.

	AAT at Diagnosis		OS		DSS	
			HR (95% CI)	*p*-Value	HR (95% CI)	*p*-Value
Overall population	Every 5-degree increment		0.964 (0.954–0.973)	<0.0001	0.959 (0.948–0.969)	<0.0001
	Temperature at Diagnosis	<Q1 [48.59]	Ref.		Ref.	
		Q1–Q3	0.909 (0.873–0.945)	<0.0001	0.902 (0.865–0.942)	<0.0001
		>Q3 [62.69]	0.873 (0.834–0.914)	<0.0001	0.854 (0.813–0.897)	<0.0001
	Temperature at Diagnosis	≤53.5	Ref.		Ref.	
		>53.5	0.889 (0.860–0.918)	<0.0001	0.873 (0.843–0.904)	<0.0001
Adenocarcinoma Subgroup	Every 5-degree increment		0.968 (0.955–0.980)	<0.0001	0.960 (0.947–0.974)	<0.0001
	Temperature at Diagnosis	<Q1 [48.59]	Ref.		Ref.	
		Q1–Q3	0.924 (0.879–0.972)	0.0023	0.917 (0.869–0.968)	0.0018
		>Q3 [62.69]	0.893 (0.842–0.947)	0.0002	0.867 (0.814–0.924)	<0.0001
	Temperature at Diagnosis	≤53.5	Ref.		Ref.	
		>53.5	0.902 (0.864–0.941)	<0.0001	0.879 (0.841–0.920)	<0.0001
Squamous Subgroup	Every 5-degree increment		0.959 (0.944–0.975)	<0.0001	0.958 (0.941–0.975)	<0.0001
	Temperature at Diagnosis	<Q1 [48.59]	Ref.		Ref.	
		Q1–Q3	0.903 (0.845–0.964)	0.0023	0.897 (0.836–0.963)	0.0026
		>Q3 [62.69]	0.852 (0.791–0.918)	<0.0001	0.843 (0.778–0.913)	<0.0001
	Temperature at Diagnosis	≤53.5	Ref.		Ref.	
		>53.5	0.877 (0.832–0.924)	<0.0001	0.871 (0.824–0.922)	<0.0001

Predictors: Temperature, Age (Continuous), Sex, Race, Stage, Histology, Grade, Primary Site, and Insurance Status.

**Table 3:** Characteristics of gastric cancer cohort according to temperature groups (n = 20,533 patients).

		≤53.5	>53.5	Overall	*p*-Value
	N	8025 (39.1)	12,508 (60.9)	20,533 (100%)	
Age	Mean/Std/N	66.87/13.29/8025	68.51/13.31/12,508	67.87/13.33/20,533	<0.001
Sex	Male	5399 (67.3%)	8272 (66.1%)	13,671 (66.6%)	0.090
	Female	2626 (32.7%)	4236 (33.9%)	6862 (33.4%)	
Race	Non-Hispanic White	4510 (56.2%)	7366 (58.9%)	11,876 (57.8%)	<0.001
	Non-Hispanic Black	1612 (20.1%)	2221 (17.8%)	3833 (18.7%)	
	Hispanic	1041 (13.0%)	1186 (9.5%)	2227 (10.8%)	
	Other	862 (10.7%)	1735 (13.9%)	2597 (12.6%)	
Marital Status	Married	4517 (56.3%)	6919 (55.3%)	11,436 (55.7%)	0.172
	Single	3508 (43.7%)	5589 (44.7%)	9097 (44.3%)	
Insurance Status	Insured	3966 (49.4%)	6144 (49.1%)	10,110 (49.2%)	0.844
	Uninsured	212 (2.6%)	344 (2.8%)	556 (2.7%)	
	Unknown	3847 (47.9%)	6020 (48.1%)	9867 (48.1%)	
Primary Site	C160 Cardia, NOS	2795 (34.8%)	4212 (33.7%)	7007 (34.1%)	0.003
	C161 Fundus of stomach	309 (3.9%)	446 (3.6%)	755 (3.7%)	
	C162 Body of stomach	605 (7.5%)	979 (7.8%)	1584 (7.7%)	
	C163 Gastric antrum	1504 (18.7%)	2487 (19.9%)	3991 (19.4%)	
	C164 Pylorus	252 (3.1%)	325 (2.6%)	577 (2.8%)	
	C165 Lesser curvature of the stomach, NOS	585 (7.3%)	1066 (8.5%)	1651 (8.0%)	
	C166 Greater curvature of the stomach, NOS	321 (4.0%)	460 (3.7%)	781 (3.8%)	
	C168 Overlapping lesion of the stomach	516 (6.4%)	769 (6.1%)	1285 (6.3%)	
	C169 Stomach, NOS	1138 (14.2%)	1764 (14.1%)	2902 (14.1%)	
Site	Stomach	5198 (64.8%)	8264 (66.1%)	13,462 (65.6%)	0.056
	Esophagus GE Junction	2827 (35.2%)	4244 (33.9%)	7071 (34.4%)	
Stage	Localized	2136 (26.6%)	3472 (27.8%)	5608 (27.3%)	0.003
	Regional	2537 (31.6%)	4112 (32.9%)	6649 (32.4%)	
	Distant	3352 (41.8%)	4924 (39.4%)	8276 (40.3%)	
Grade	I/II	2682 (33.4%)	4327 (34.6%)	7009 (34.1%)	<0.001
	III/IV	4160 (51.8%)	6644 (53.1%)	10,804 (52.6%)	
	Unknown	1183 (14.7%)	1537 (12.3%)	2720 (13.2%)	

**Table 4:** Multivariate analysis of AAT as a predictor of OS and DSS in the gastric cancer cohort.

Temperature at Diagnosis		OS		DSS	
		Hazard Ratio (95% CI)	*p*-Value	Hazard Ratio (95% CI)	*p*-Value
Every 5-degree increment		0.961 (0.952–0.971)	<0.0001	0.955 (0.945–0.965)	<0.0001
AAT at Diagnosis (Categorical)	<Q1 [49.5667]	Ref.		Ref.	
	Q1–Q3	0.904 (0.871–0.938)	<0.0001	0.886 (0.851–0.923)	<0.0001
	>Q3 [62.5750]	0.869 (0.833–0.907)	<0.0001	0.855 (0.816–0.895)	<0.0001
The temperature at Diagnosis (Categorical—Binary)	≤53.5	Ref.		Ref.	
	>53.5	0.875 (0.848–0.903)	<0.0001	0.858 (0.829–0.887)	<0.0001

Predictors: Temperature, Age (Continuous), Sex, Race, Stage, Histology, Grade, Primary Site, and Insurance Status.

## Data Availability

Data are contained within the article.
